# Aging and skeletal muscle force control: Current perspectives and future directions

**DOI:** 10.1111/sms.14207

**Published:** 2022-07-28

**Authors:** Jamie Pethick, Matthew J. D. Taylor, Stephen D. R. Harridge

**Affiliations:** ^1^ School of Sport, Rehabilitation and Exercise Sciences University of Essex Essex UK; ^2^ Centre for Human and Applied Physiological Sciences King's College London London UK

**Keywords:** aging, complexity, force control, force steadiness, motor unit, muscle, physical activity

## Abstract

During voluntary muscle contractions, force output is characterized by constant inherent fluctuations, which can be quantified either according to their magnitude or temporal structure, that is, complexity. The presence of such fluctuations when targeting a set force indicates that control of force is not perfectly accurate, which can have significant implications for task performance. Compared to young adults, older adults demonstrate a greater magnitude and lower complexity in force fluctuations, indicative of decreased steadiness, and adaptability of force output, respectively. The nature of this loss‐of‐force control depends not only on the age of the individual but also on the muscle group performing the task, the intensity and type of contraction and whether the task is performed with additional cognitive load. Importantly, this age‐associated loss‐of‐force control is correlated with decreased performance in a range of activities of daily living and is speculated to be of greater importance for functional capacity than age‐associated decreases in maximal strength. Fortunately, there is evidence that acute physical activity interventions can reverse the loss‐of‐force control in older individuals, though whether this translates to improved functional performance and whether lifelong physical activity can protect against the changes have yet to be established. A number of mechanisms, related to both motor unit properties and the behavior of motor unit populations, have been proposed for the age‐associated changes in force fluctuations. It is likely, though, that age‐associated changes in force control are related to increased common fluctuations in the discharge times of motor units.

## 1 INTRODUCTION

**FIGURE 1:**
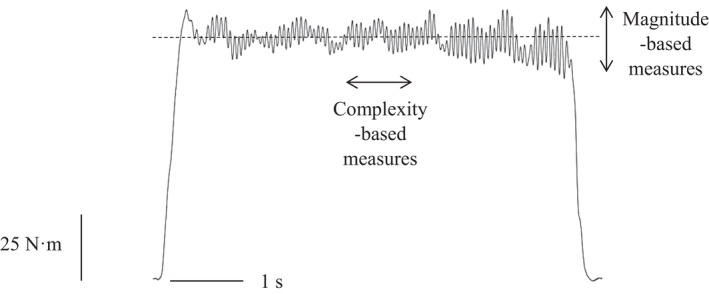
Raw force output from an isometric knee extension contraction performed at 40% of participants' maximal voluntary contraction (MVC). Note the constant fluctuations above and below the imposed target. These fluctuations have typically been quantified according to their magnitude, using measures such as the standard deviation and coefficient of variation (which, in this case are 3.9 N·m and 3.9%, respectively), and more recently according to their temporal structure, using complexity‐based measures such as approximate entropy, sample entropy and detrended fluctuation analysis α (which, in this case are 0.58, 0.54 and 1.16, respectively)

The motor unit, consisting of a single motor neuron and the muscle fibers it innervates, is the basic functional unit of the neuromuscular system responsible for transducing synaptic input from the central nervous system into force and movement.^1^, ^2^ Through the processes of recruitment and derecruitment of motor units, and modulation of their discharge rates, muscle force is controlled and modified according to task demands.^3^ This force is not, however, smooth and completely accurate; rather, it constantly fluctuates around the required target^4^, ^5^, ^6^ (Figure 1). The presence of such constant fluctuations has significant implications for task performance in a variety of contexts and relative force levels.^7^, ^8^

Age‐related changes to motor units have a profound effect on both maximal force generating capacity (i.e., strength) and force control and, consequently, task performance.^5^, ^9^ These changes, including a net loss of motor units,^3^ motor unit remodeling,^10^ alterations in discharge rates^11^ and alterations in common synaptic input to motor neurons,^12^ compromise the ability to generate task‐relevant and precise levels of force.^5^, ^13^ Numerous studies have demonstrated differences in both the magnitude^14^, ^15^ and, more recently, the temporal structure (i.e., “complexity”)^16^, ^17^ of force fluctuations between old and young adults.

From a functional perspective, the age‐associated alterations in force fluctuations have been shown to contribute to the reduced ability of older adults to perform activities of daily living (ADLs), including balance, mobility, and object manipulation.^18^, ^19^, ^20^ Moreover, it has been suggested that, at least in the early stages of getting older, declines in functional capacity are more closely related to impaired force control than a reduced capacity to generate maximal force.^3^ In recent years, there has been a significant push to increase our understanding of how force control changes with age given that force fluctuations appear to contribute to many of the most functionally relevant performance decrements seen with aging.^21^

The purpose of this review is to provide a comprehensive examination of age‐associated changes in force control. This examination first necessitates a description of the measurement and quantification of force fluctuations; with the latter being of critical importance given that this review is the first to address changes in both the magnitude and complexity of force fluctuations. We then provide empirical evidence regarding age‐associated changes in force fluctuations and their functional implications, before discussing whether this is an inherent component of the aging process (or whether it reflects an interaction of aging and inactivity^22^), what interventions might reverse these changes and the potential underpinning mechanisms.

## 2 MEASUREMENT AND QUANTIFICATION OF FORCE FLUCTUATIONS

Force fluctuations are typically assessed during submaximal isometric (or sometimes anisometric) contractions at an imposed target force.^5^, ^23^ During such contractions, the exerted force will fluctuate around the imposed target (Figure 1). These fluctuations have traditionally been regarded as “noise” and quantified according to their *magnitude*, using metrics such as the standard deviation (SD) or coefficient of variation (CV).^5^ Such magnitude‐based metrics provide an index of the degree of deviation from a fixed point within a time‐series and assume that fluctuations are random and independent.^4^ The SD of isometric force linearly scales with respect to force^24^ and provides a measure of the absolute variability in an output. In order to better accommodate differences in strength between subject groups, as is evident with young and old adults,^25^ the SD can be normalized to the mean force and expressed as the CV.^9^ Increases in both the SD and CV are interpreted as decreased force steadiness (i.e., increased magnitude of variability).

Advances in analytical techniques have led to the recognition that fluctuations in muscle force are neither random nor independent but rather possess a statistically irregular temporal structure or “*complexity*”.^4^ Complexity metrics characterize the moment‐to‐moment relationship between successive points in a time‐series^26^; thereby characterizing how an output evolves over time. Moreover, they quantify irregularity, time irreversibility and long‐range fractal correlations; properties that magnitude‐based metrics cannot quantify. Thus, complexity‐based metrics provide information additional to, and distinct from, magnitude‐based metrics, and it has been argued that the two approaches should be used in conjunction in order to provide a more complete understanding of force control.^4^, ^27^ Measures of complexity reflect the adaptability of force production,^28^ defined as the ability to adapt force output rapidly and accurately in response to task demands.^16^ It has been suggested that the magnitude and complexity of force fluctuations may differ in their functional significance.^29^, ^30^

Complexity measures are derived from the field of non‐linear dynamics and include those related to information theory (e.g., entropy statistics), which quantify the apparent regularity or randomness of an output, and those related to fractal geometry, which quantify long‐range correlations within an output.^27^ An important caveat when applying these metrics is that no single statistical measure can fully capture the complexity of physiological outputs, and, as such, it is recommended that multiple metrics, which probe subtly different aspects of the output are used.^28^

Complexity in muscle force has typically been quantified using approximate entropy (ApEn),^26^ sample entropy (SampEn),^31^ and detrended fluctuation analysis (DFA)^32^ (Figure 1). ApEn and SampEn are regularity statistics, which evaluate time‐series for patterns that recur. They quantify a continuum ranging from 0 to 2, with low values indicating more regularity or less complexity and higher values indicating low regularity and high complexity.^26^ They differ in that SampEn does not count self‐matches when evaluating for recurring patterns; a characteristic purported to give it greater relative consistency.^31^ Importantly, high entropy values, such as that of white noise, are not necessarily *physiologically* complex. As such, metrics such as DFA, which can estimate the temporal fractal scaling and differentiate the noise color of an output, are necessary to fully characterize physiologic complexity. The DFA α exponent theoretically ranges from ~0.5 to ~1.5 and differentiates outputs that are random (i.e., white noise, α = 0.5), possess statistically self‐similar fluctuations (i.e., pink or 1/f noise, α = 1.0) or are Brownian in nature (i.e., with long‐term memory, α = 1.5).^28^

## 3 AGE‐ASSOCIATED CHANGES IN FORCE FLUCTUATIONS

**FIGURE 2:**
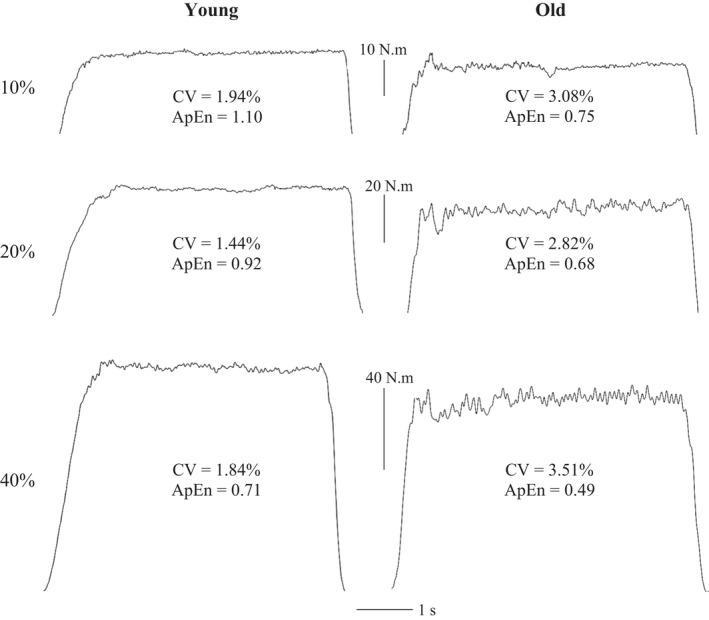
Raw force outputs from a young (age 21) and an old (age 60) adult during isometric knee extension contractions at 10%, 20% and 40% MVC. The output of the old adult is characterized by a greater magnitude of variability, as measured by the CV, and lower complexity, as measured by ApEn. Unpublished data (University of Essex ethics ref. ETH2021‐0394)

There is strong evidence that older adults (aged > 60 years) exhibit a greater magnitude of force fluctuations than young adults (aged ~20–30 years), which is interpreted as a decrease in force steadiness (Figure 2). Indeed, a recent meta‐analysis found a significant pooled effect size of 0.67 for the effect of age on force steadiness.^23^ There is also growing evidence demonstrating that older adults (who are typically inactive or have moderately active lifestyles) exhibit lower complexity in force fluctuations than their younger counterparts,^16^, ^33^ with this being interpreted as a decrease in the adaptability of force output. This loss of complexity exhibited by older adults is typically characterized by decreases in entropic measures (i.e., toward 0), indicating increased regularity, and increases in DFA α (i.e., toward 1.5), indicating increasingly Brownian fluctuations (Figure 2). It is important to note, however, that the exact nature of the age‐related changes in the magnitude and complexity of force fluctuations are dependent on a number of factors, including the muscle group performing the task, the intensity of the contraction, the type of contraction and whether the task is performed with an additional cognitive load.

### 3.1 Muscle group

The various muscle groups of the body are characterized by physiological differences (e.g., muscle fiber type distribution and contractile properties^34^; motor unit innervation ratio^35^) and functional differences (e.g., fine or gross motor control). Age‐associated differences in the magnitude and complexity of muscle force fluctuations are evident in both small muscles of the upper limb, associated with fine motor skills, and large muscles of the lower limb, associated with locomotion and posture. There do appear, however, to be some exceptions to the loss‐of‐force control.

The initial studies investigating age‐associated changes in force control were conducted in the muscles of the hand. Galganski et al.^14^ observed a greater magnitude of index finger abduction (i.e., first dorsal interosseous) force fluctuations in older adults during contractions between 2.5% (CV, old vs. young = 11.0 ± 1.8 vs. 6.6 ± 0.5%) and 50% maximum voluntary contraction (MVC; CV, 3.9 ± 0.2 vs. 2.9 ± 0.2%); an observation subsequently confirmed by others.^15^, ^36^ Indeed, the index finger abductors are one of the muscle groups most affected by age‐associated changes in force fluctuations, with a meta‐analysis finding a significant pooled effect size of 0.79, the largest effect size of any muscle group included in the analysis.^23^ From a more functional perspective, older adults exhibit greater absolute and relative variability during bi‐ and tri‐digit finger pinch tasks.^37^, ^38^, ^39^ Moreover, the age‐associated increase in tri‐digit pinch force variability was greater in the non‐dominant limb,^38^ suggesting that habitual daily use can effect age‐associated changes in force control. Taken together, these findings indicate a loss of fine force control.

There is also evidence of an age‐associated increase in the magnitude of force fluctuations in the knee extensors.^23^ Such a loss‐of‐force control in the knee extensors is of importance for locomotion and balance. Indeed, older adults, particularly those with a history of falling,^18^ have been demonstrated to exhibit a greater CV of force during low‐intensity contractions (≤10%MVC).^40^ Interestingly, in one all‐female study, age‐associated differences in the magnitude of force fluctuations were only found at 80% MVC and not at low‐ or moderate‐intensity contractions,^41^ and in another, no differences were observed at all,^42^ suggesting potential sex differences in the loss‐of‐force control.

The muscles responsible for force control about the ankle and, therefore, involved in regulating control of posture during standing and of stance and swing during gait, are also affected by the age‐associated loss‐of‐force control. A greater CV of force fluctuations has been consistently observed in older adults during low‐intensity plantarflexion contractions.^43^, ^44^, ^45^ With regards to dorsiflexion, Tracy^43^ observed no differences in the CV between young and old adults, which was in line with the lack of difference in maximal force between the two age groups. Nevertheless, further research has demonstrated a greater magnitude of force fluctuations in older adults during low‐intensity dorsiflexion contractions.^20^, ^46^

In contrast to the above, evidence suggests that force fluctuations about the elbow are less affected by age. Indeed, the elbow flexors were the only muscle group included in Oomen and van Dieën's^23^ meta‐analysis not to exhibit a significant effect of age. Several studies have found no difference in the CV of isometric elbow flexion between old and young adults during contractions performed over a large range of contraction intensities (2%–70% MVC).^47^, ^48^ Similarly, Lavender and Nosaka^49^ found no difference in elbow flexor force variability between young and old adults during contractions ranging from 30%–80% MVC either in fresh muscle or after performance of muscle damaging eccentric exercise. Further studies have, nonetheless, observed age‐related increases in elbow flexor force variability at very low contraction intensities (2.5% MVC), with this effect exacerbated when no visual feedback was provided.^50^

As with the magnitude of force fluctuations, initial studies on age‐associated changes in the complexity of force fluctuations were conducted on the first dorsal interosseous. Vaillancourt and Newell^16^ observed a significant and progressive loss of complexity, quantified as decreased ApEn and increased DFA α from young (ApEn = ~0.50, DFA α = ~1.24) to old (ApEn = ~0.42, DFA α = ~1.30) and older‐old adults (ApEn = ~0.36, DFA α = ~1.36). These findings in the first dorsal interosseous have subsequently been confirmed numerous times for contractions between 2 and 25% MVC.^51^, ^52^, ^53^ These results have been extended to bi‐digit pinch grip,^54^ knee extension contractions^33^ and ankle plantarflexion contractions.^17^ Furthermore, there appears to be a progressive decrease in ApEn in the knee extensors when comparing non‐frail, pre‐frail and frail older adults.^55^ This raises issues about how older adult populations are defined and selected for research purposes.^56^ Indeed, it has been argued that the study of older adults requires tightly defined, pre‐determined criteria in order to select adults who are “healthy”^57^ and in which their physical activity status is defined.^56^

### 3.2 Contraction intensity

In both old and young adults, the SD of isometric force of all muscle groups linearly scales with respect to contraction intensity,^4^, ^5^ referred to as signal dependent noise.^24^ It is thus greatest during maximal contractions. The CV, on the other hand, is greatest at the lowest intensities and decreases in an exponential fashion.^58^ The relationship between complexity and contraction intensity appears to be muscle group dependent, with some (e.g., first dorsal interosseous) exhibiting an inverted‐U shaped relationship^4^ and others (e.g., knee extensors) exhibiting a linearly decreasing relationship.^59^ Age‐related changes in both the magnitude and complexity of force fluctuations are heavily dependent on contraction intensity.

Initial studies on aging and force control observed a greater CV of fluctuations in older adults across all contraction intensities tested, from 5% to 50% MVC.^14^ Subsequent research, however, has demonstrated that age‐associated increases in the magnitude of force fluctuations are contraction intensity dependent, occurring primarily at intensities between 2.5% and 10% MVC.^23^, ^40^ Consistent, though smaller, differences in CV are still evident up to ~40% MVC.^23^, ^38^ For contraction intensities above 40% MVC, no significant differences in the magnitude of force fluctuations are typically observed.^23^, ^36^ This increase in the magnitude of force fluctuations at predominantly low‐intensities is particularly important, given that most ADLs, particularly those performed by older adults, only require forces of up to 20% MVC.^60^

In contrast to the magnitude of force fluctuations, the loss of complexity in force fluctuations appears to be evident across all contraction intensities so far tested. The majority of studies have only investigated low‐ to moderate‐intensity contractions, observing decreased complexity, using a variety of metrics (i.e., ApEn, SampEn, multiscale entropy and DFA α), in older adults during contractions ranging from 5%–40% MVC.^16^, ^33^, ^51^, ^54^ Challis^17^ extended these findings to high‐intensity contractions, observing lower ApEn in old adults (0.25 ± 0.07) compared to young adults (0.35 ± 0.07) during maximal plantarflexion contractions. Moreover, this loss of complexity occurred in the absence of any age‐related difference in the CV of fluctuations (old vs. young, 5.8 ± 1.4 vs. 5.7 ± 1.4%), suggesting that complexity‐based metrics could be more sensitive to subtle changes undetected by more classical magnitude‐based metrics. Unfortunately, little research has investigated the effect of aging on the complexity of force fluctuations during contractions between 40% and 100% MVC.

### 3.3 Contraction type

Many ADLs require either the maintenance and/or modulation of a specific force.^54^ Constant force (i.e., isometric) tasks have been the most prominent paradigm used to investigate age‐related changes in force fluctuations, though isometric force tracking (i.e., sine‐wave tracking) and concentric/eccentric tasks can also provide useful information.^5^, ^54^

During isometric,^14^, ^39^ sine‐wave tracking tasks^38^ and concentric and eccentric tasks,^15^, ^36^ older adults exhibit an increased magnitude of force fluctuations compared to young adults. This age‐associated increase in the magnitude of force fluctuations is typically greater during sinusoidal, concentric and eccentric tasks than during isometric contractions.^36^, ^38^ Furthermore, eccentric contractions appear to be less steady than concentric contractions in old, but not young adults,^15^, ^36^ which could have functional implications when performing activities such as descending stairs. This difference in force fluctuations between isometric and anisometric contractions has been attributed to differences in recruitment thresholds and discharge rates between contraction types.^5^ It has been estimated that the discharge of a single motor unit can account for ~30% of the force fluctuations during slow anisometric contractions, but only 4% during position maintenance tasks.^61^, ^62^

During isometric tasks, older adults exhibit lower complexity compared to young adults.^16^, ^17^ In contrast to this, older adults have been found to demonstrate greater complexity than young adults during sine‐wave tracking tasks.^16^ For example, Sosnoff and Newell^52^ found ApEn values of 0.48 and 0.37 for old and young adults during an isometric task at 10% MVC, and values of 0.16 and 0.21 during a sine‐wave task. This supports the “bidirectional theory of complexity”, in which change is dependent on task dynamics.^63^ In tasks where the dynamic is constant (i.e., isometric), more complexity is required to maintain optimal output. For such tasks there will be a decrease in complexity with increasing age because, in order to realize the goal of no motion, additional degrees of freedom must be introduced, which older adults generally find more difficult to accomplish.^16^ However, in tasks where the dynamic is oscillatory (i.e., sine‐wave), less complexity is required to closely track oscillations and reduce error. The observed increase in complexity with aging is due to older adults having difficulty reducing the dimension of their output to a lower dimension than the resting state of the system.^16^, ^63^

### 3.4 Dual‐tasking

Many ADLs involve simultaneous performance of cognitive and motor tasks.^64^ The variability and complexity of force fluctuations in young adults can be affected by the addition of a cognitive task to a motor task.^65^ As aging is associated with declines in both cognitive and motor function,^66^ adding a cognitive task to a motor task could have important functional implications.

Voelcker‐Rehage et al.^67^ found that older adults exhibited an increase in the CV of bi‐digit pinch force fluctuations during contractions at 20% MVC when an additional cognitive (memory) task was imposed (CV with no cognitive task = 2.14 ± 1.23; with cognitive task = 3.14 ± 2.00%), whereas young adults maintained performance equally well in both conditions (CV with no cognitive task = 1.40 ± 0.62; with cognitive task = 1.53 ± 0.74%). The increase in force fluctuations of older adults was also directly related to the difficulty of the cognitive task and increased further when they made a mistake in the task. Further studies have demonstrated this increased variability with additional cognitive demand is also evident in larger muscle groups (i.e., elbow flexors, ankle dorsiflexors) and is particularly evident during low‐intensity contractions.^64^, ^66^

## 4 FUNCTIONAL IMPLICATION OF CHANGES IN FORCE FLUCTUATIONS

An impaired ability to control force will result in a neuromuscular response that is insufficient to withstand a perturbation or adequately compensate when performing a task.^3^ It is, therefore, no surprise that the above‐described age‐associated loss‐of‐force control is likely to contribute to reduced function in a wide range of ADLs, involving both muscles of the upper and lower limbs. Indeed, it appears that the age‐associated loss‐of‐force control may be as, if not more, important for functional capacity than the loss of maximal strength.^3^, ^8^

### 4.1 Clinical measures of functionality

Balance, locomotion and manual dexterity represent three fundamental motor skills^68^; the performance of which can be clinically measured using tests of standing balance, walking speed, chair stand time and time taken to complete a pegboard task.^19^, ^69^, ^70^ Initial research failed to demonstrate a link between knee extensor force variability and clinical indices of balance and locomotion,^71^ though can be criticized for measuring force fluctuations at 50% MVC, a contraction intensity at which differences between old and young subjects are less evident.^23^ Subsequent studies, which have measured force fluctuations at the lower intensities typical of ADLs,^60^ have found ample evidence linking force fluctuations to clinical indices of functionality.

Kouzaki and Shinohara^44^ were the first to demonstrate a link between force fluctuations and balance during quiet standing, observing a significant positive correlation (*r* = 0.455) between the CV of plantarflexion force during contractions at ≤5% MVC and the CV of foot center of pressure displacement (a measure of postural sway). This relationship was observed for both young and old adults, though with the old adults exhibiting significantly greater CVs of both force fluctuations and center of pressure displacements. Similarly, a correlation between plantarflexion force variability (at 20% MVC) and postural sway has been observed in older women when standing on an unstable surface.^72^ Moreover, in this study there were no correlations observed between postural sway and MVC. The muscles crossing the ankle joint are not the only ones involved in maintaining balance. Davis et al.^20^ found that high postural sway when standing on a foam surface with eyes open was mediated by a greater magnitude of force fluctuations not only in the plantarflexors and dorsiflexors, but also the hip abductors.

Increased postural sway and sensorimotor variability have been proposed to be major risk factors for falls in older adults.^20^ In support of this, older adults with a history of falling have been demonstrated to exhibit greater variability during both isometric and eccentric knee extension contractions than older adults with no history of falling and young adults.^18^ Taken together, the above findings seemingly link force control, postural sway and falls. In contrast, a recent systematic review found no conclusive evidence of an association between strength and falls.^73^

With regards to locomotion, a correlation between muscle force accuracy (defined as the difference between the exerted and target forces) during eccentric knee extensor contractions at 50% MVC and measures of mobility in older adults with a history of falling has been observed.^74^ Importantly, this correlation was evident for each of the mobility measures tested: the 6‐min walk test, timed up and go test, and timed stair ascent and descent tests. Similarly, higher variability of isometric knee extension force at 50% MVC has been found to predict a slower speed of chair rise time and lower stair climbing power in older women.^75^ Interestingly, these correlations were evident at a much higher proportion of MVC than those between force control and balance (≤20% MVC), suggesting that different functional activities have different force control requirements. In support of this assertion, Mani et al.^8^ found no correlation between ankle plantarflexor and dorsiflexor force variability at 10% and 20% MVC and various tests of mobility (e.g., 400 m walk time, 10 m walk at preferred and maximal speeds, chair stand time) in older adults. The discharge properties of motor units, which are important mediators of force fluctuations,^15^ were, however, correlated with mobility.

For manual dexterity tasks, moderate correlations have been observed between performance on a pegboard task and index finger abduction, bi‐digit pinch and wrist extensor forces across both young and old adults.^19^, ^39^ Moreover, the estimated variance in common synaptic input, postulated to be the main determinant of force fluctuations,^76^ is significantly associated with time to complete a pegboard task only in old adults.

### 4.2 Other indices of functionality

As many ADLs have both motor and cognitive components, the greater variability exhibited by older adults during dual‐task conditions could have significant functional effects. An innovative study by Lodha et al.^77^ investigated force control during reactive driving, a task that involves responding to unexpected stimuli with accurate and consistent movements. Subjects performed a sinusoidal tracking task with the ankle dorsiflexors, along with a reactive driving task involving responding to unexpected brake lights. Older adults exhibited greater variability during the force tracking task and in force applied to the brake pedal during the driving task. Importantly, the poorer performance of older adults in the reactive driving task was significantly correlated with force (*r* = 0.48), but not with strength.

Other ADLs involve the maintenance of low levels of force for prolonged periods of time^60^ and, as such, fatigue represents a significant functional limitation. Variance in endurance for older adults during a submaximal isometric task has been found to be most closely associated with age, force variability and strength.^70^ Among older adults, adding the baseline variability and complexity of knee extensor contractions to gender and obesity increased the explanatory power of a regression model for endurance time from 16.2% to 49%.^78^

## 5 INTERVENTIONS TO REVERSE AGE‐ASSOCIATED CHANGES IN FORCE FLUCTUATIONS

To determine whether aging processes, inactivity processes or a combination of both contribute to increased torque complexity requires investigation of physically active/exercising older people. The fact that the detrimental age‐associated changes in force control appear to be reversible, at least to a certain extent suggests that this is not solely an aging phenomenon. Acute interventions such as skilled movement training^79^ and various forms of exercise, including strength training^80^ and Tai Chi,^81^ have been demonstrated to improve force steadiness and complexity. Moreover, these interventions seem to be effective in a very short period of time (as little as ~2 weeks).^82^, ^83^ There is limited evidence, though, regarding how, or if, these improvements in steadiness and complexity affect functional performance,^9^ particularly with regards to tasks involving the lower limbs. Indeed, Barbosa et al.^84^ demonstrated that force steadiness training in older women decreased the magnitude of variability in plantarflexion force, but this was insufficient to affect postural sway.

Practice of a skilled motor task has been demonstrated to improve both force steadiness and manual dexterity. In Ranganathan et al.,^79^ older adults were required to manipulate two metal balls in the palm of their hand twice a day for 8 weeks. Following this training, subjects exhibited a decrease in the SD of tri‐digit pinch force during contractions at intensities ≤20% MVC, which was accompanied by a significant decrease in the time taken to perform a pegboard task. The training also resulted in an increase in motor neuron excitability, which the authors suggested may have contributed to the improved force control. Similarly, older adults who practiced a pegboard task for only 2 weeks demonstrated improved performance in that task, along with decreases in the variability of bi‐digit pinch and index finger abduction force.^82^

With regards to strength training, a decrease in the magnitude of force fluctuations in the first dorsal interosseous of older adults has been observed during slow concentric and eccentric contractions following 2 weeks of light load training, consisting of lifting and lowering a load of 10% MVC.^83^ Interestingly, a further 4 weeks of heavy load training at 70% MVC resulted in no further improvement in force control. Further studies have observed improvements in force control in older adults following both low‐ and high‐intensity training loads. Laidlaw et al.^85^ observed similar decreases in the SD and CV of index finger abduction force at contraction intensities ≤20% MVC following 4 weeks of training at 10% or 80% MVC and no change in a control group. Similarly, Keen et al.^80^ found a decrease in CV after 4 weeks of a 12‐week training program performed at 80% MVC. In contrast, this same training had no effect on the CV of force in young adults. Taken together, these studies indicate that improvements in force steadiness in older adults can be seen after training for only a short period of time (<4 weeks) at a low‐intensity. This suggests that such improvements in force control are likely mediated by adaptations in motor unit recruitment and discharge characteristics, rather than increased strength per se.

Older males (aged 70–80) who underwent 6 weeks of upper body strength training (consisting of dumbbell biceps curls, wrist flexions and wrist extensions) in just one limb, decreased the CV (9.9 ± 13.1 to 6.0 ± 6.2%) and increased the SampEn (0.16 ± 0.13 to 0.27 ± 0.19) of tri‐digit pinch force in the trained limb.^86^ Moreover, there was also a significant effect of training on complexity, but not variability, in the untrained limb. These results have several important implications. Firstly, that the training exercises differed from the testing tasks shows that improvements in fine motor control can be gained through the performance of more global, gross motor tasks involving larger muscle mass. Secondly, that complexity, but not variability, was affected in the untrained limb provides further evidence that complexity measures may be more sensitive to change than variability measures. And finally, that complexity was increased in the trained and untrained limbs suggests that both muscular and neural adaptations play mechanistic roles in the improved force control.

Studies on strength training in the lower body have, however, found more equivocal results. Bellew,^87^ for example, observed a significant increase in knee extensor MVC but no effect on either the SD or CV of force fluctuations during isometric contractions at 30%, 60% and 90% MVC following 12 weeks of high‐intensity strength training in older adults. Similarly, 16 weeks of low‐intensity knee extensor training elicited improvements in MVC but had no effect on isometric steadiness.^88^ These studies provide further evidence that muscle strength and force steadiness are dissociated. Training‐induced increases in knee extensor force control have, nevertheless, been observed. Kobayashi et al.^89^ observed that 8 weeks of low‐intensity training increased knee extensor MVC and decreased the magnitude of force fluctuations at 10%, 30% and 65% MVC. A similar decrease in the magnitude of force fluctuations was observed in the elbow flexors, but in the absence of an increase in MVC; further highlighting that improvements in force steadiness are independent of increases in strength.

Forms of exercise other than structured resistance training can also improve force control. Tai Chi is a low‐ to moderate‐intensity activity that involves a series of slow, fluid movements of the body with the aim of enhancing balance and stability. Christou et al.^81^ observed a significant increase in knee extensor MVC and a decrease in the CV of force in older adults following 20 weeks of Tai Chi training. Similarly, it has been observed that Tai Chi training can improve the ability to exert accurate forces when making arm movements, despite Tai Chi not significantly loading muscles in the upper body.^90^

Taken together, these results indicate that indices of muscle force steadiness and complexity fall under the classification of variables that are age‐dependent but malleable by exercise.^22^ An interesting implication of this is that the loss of muscle force control might not simply be an inherent age‐associated phenomenon. Rather, it may relate to amount of use/disuse throughout the lifetime^22^ (discussed further below in “Future research directions”). Two observations support this contention. Firstly, the age‐associated increase in tri‐digit pinch force variability has been demonstrated to be greater in the non‐dominant hand, which is subject to less habitual daily use,^38^ and secondly, experimentally induced physical inactivity (brought about by limb immobilization) has been demonstrated to significantly increase both ankle plantarflexor and knee extensor force variability.^91^

## 6 MECHANISMS UNDERPINNING AGE‐ASSOCIATED CHANGES IN FORCE FLUCTUATIONS

As motor units transduce synaptic input from the central nervous system into muscle force, changes in their properties and the input to them are responsible for force fluctuations. Aging is characterized by a number of detrimental effects on the motor unit, including a net loss of motor units, changes to the morphology and properties of existing motor units, and altered input from peripheral, spinal and supraspinal centers^9^; all of which have been postulated to contribute to the age‐associated loss‐of‐force control.

### 6.1 Motor unit properties

Both simulation and experimental data have demonstrated that weaker muscles exhibit greater force variability.^58^ Accordingly, Sosnoff and Newell^51^ concluded that age‐associated changes in force variability and complexity described above are more fundamentally due to the association between strength and force control, rather than chronological age. In support of this, knee extensor MVC and force complexity are both decreased in frail compared to non‐frail older adults.^55^ Further studies have, however, found that increased variability in older adults is dissociated from the decline in strength.^47^ Moreover, training‐induced increases in older adults' strength have not always been associated with improvements in force control.^87^, ^88^ These results, therefore, suggest that muscle strength per se is not responsible for the age‐associated loss‐of‐force control; rather, it is more likely that specific properties of motor units (i.e., their recruitment, discharge rates, twitch forces) and the input to them that contribute to the age‐associated loss‐of‐force control.

The loss‐of‐force complexity with advancing age has been speculated to relate to the remodeling of motor unit populations,^17^ in that smooth control of force is negatively affected by having a lower number of motor units, but with each containing more fibers. This remodeling involves apoptosis of spinal motor neurons, leading to a decline in the number of motor units but a partial reinnervation of surviving motor units.^10^ Consequently, older adults recruit fewer, but larger, motor units when generating a relative level of force. As such, the spike‐triggered average force of motor units in the first dorsal interosseous has been demonstrated to be greater in adults.^14^ The effect of aging on force control is particularly evident at low forces, where each motor unit has a larger contribution to net force.^92^ Fluctuations in motor unit force when the unit is first recruited and discharging at low rates are greater in older adults^93^ and this has been speculated to contribute to the age‐associated difference in force fluctuations at low‐intensities.

Simulation studies, have, however, indicated that increases in the amplitude of motor unit twitch forces have a negligible effect on force fluctuations.^94^ Consistent with this, 4 weeks of strength training decreased the CV of older adults' first dorsal interosseous force but had no effect on mean motor unit force.^80^ That the decrease in the magnitude of force fluctuations occurred within 4 weeks suggests that the mechanism responsible was of neural origin, as muscle fiber hypertrophy and increases in motor unit force take longer to occur.^95^ Furthermore, McNeil et al.^96^ observed that despite a significant loss of motor units in the four decades between age 25 and 65, muscle function was not reduced until after age 80. These findings suggest that older adults having fewer larger motor units does not, in fact, contribute to age‐associated differences in force fluctuations.

A further speculated mechanism is a difference in motor unit discharge properties between young and old adults. Simulation studies have indicated that varying the CV of motor unit discharge has a more pronounced effect on force fluctuations than reducing the number of motor units.^94^ Experimental studies have found that the discharge rates of older adults are lower^11^, ^97^ and more variable^98^; with further studies finding these variables to be associated with greater force variability in older adults.^15^, ^45^ However, increased force variability has been observed in the absence of any difference in the variability of discharge rates,^99^ just as decreased discharge rates have been found in the absence of any difference in the magnitude of variability.^100^ Moreover, Castronovo et al.^12^ found no association between age‐associated differences in force variability and either motor unit discharge rate or variability. These factors are mainly due to independent input to each motor neuron and, as discussed below, it is common input to motor neurons that is the main determinant of force fluctuations.^76^

### 6.2 Neural input to motor neurons

Motor neurons receive both independent and common synaptic input from a multitude of sources.^21^ The independent inputs are effectively filtered out, while the common input is transmitted to the output of the motor neurons.^76^ This common synaptic input drives the discharge rates of motor neurons at a common low frequency (necessitating a degree of motor unit synchronization) and represents the effective neural drive to muscle.^101^ Consequently, common synaptic input has been postulated to be the main determinant of force fluctuations.^76^ Indeed, it has been demonstrated that the force output of a population of motor units is highly coherent with the common component of the cumulative motor unit spike train.^102^

Common modulation of motor unit activity can be assessed by either determining the level of synchronization between the discharge times of motor units (a time‐domain measure) or by performing a coherence analysis (a frequency‐domain measure).^5^ Computer simulations indicate that increased motor unit synchronization leads to increased force fluctuations,^103^ though experimental studies have failed to find any difference in motor unit synchronization between old and young adults.^99^, ^104^ Coherent motor unit activity may be critical for fine force control, though excessive coherence is viewed as maladaptive.^9^ Older adults demonstrate a greater strength of coherent motor unit activity and, therefore, a high amplitude of common input, than young adults during low‐intensity contractions of the first dorsal interosseous.^105^ An earlier study on the same subjects found a greater magnitude of force fluctuations in older adults.^99^ Recently, Castronovo et al.^12^ demonstrated significant positive relationships between motor unit coherence (and the amplitude of common input fluctuations) in the tibialis anterior and age (*R* ^2^ = 0.5, *P* < 0.01), and between force variability and common input fluctuations (*R* ^2^ = 0.59, *P* < 0.01). Furthermore, differences in the magnitude of force fluctuations in the wrist extensors between voluntary and evoked contractions indicate that variance in common synaptic input is greater for older adults than young adults.^106^ Taken together, these findings indicate changes in common synaptic input to muscle likely explain a large part of the impaired force control exhibited by older adults. It must be noted, though, that the source of this increased common synaptic input with aging remains to be determined.^12^

One source of synaptic input received by motor neurons arises from neuromodulatory pathways from the brainstem.^21^ Monoaminergic projections from the brainstem can either increase or decrease the excitability of motor neurons.^107^ As such, degeneration of neurotransmitter systems system may contribute to age‐associated changes in common synaptic input to motor neurons and, consequently, declines in force control.^108^ Aging is characterized by a decline in D_2_ dopamine receptors and lower concentrations of serotonin.^108^ No studies have been conducted on force control and these neurotransmitters in older adults, though studies on young adults have pointed to the potential role they have. For example, antagonism of the D_2_ receptor increases force variability during low‐and moderate‐intensity elbow flexion contractions^109^; and ingestion of a selective serotonin reuptake antagonist improves force control, while selective serotonin reuptake inhibitors decrease force control.^110^

## 7 FUTURE RESEARCH DIRECTIONS

The evidence presented above has demonstrated that it is possible to reverse decrements in force control exhibited by older adults with various types of acute exercise training. Of greater interest and significance, though, is whether the decrement in force control in later life can be attenuated (or even prevented in the first place) and, therefore, whether the accompanying decrease in functional performance can be attenuated.

The necessity to be physically active in order to maintain health and physical function throughout the lifetime is well established.^111^ Indeed, it has been postulated that a certain threshold of physical activity throughout the lifespan is necessary in order to age optimally and be subject to a steady and controlled diminution of physiological function, whereas activity below this threshold results in aging contaminated by the deleterious effects of inactivity.^112^ Given the contrasting effects of physical activity and inactivity on physiological function, it is vital to select appropriate participants in order to study the inherent aging process. As such, it has been suggested that lifelong active adults (those who regularly exercise up to those who could be termed “master athletes”) represent the ideal biological model to study inherent aging, as the deleterious effects of inactivity are absent.^112^, ^113^

Lifelong physical activity (both endurance and resistance training) has been demonstrated to slow the progression of age‐associated effects on muscle output (i.e., strength),^114^ to slow the decrease in efferent drive to muscle^115^ and to enhance the remodeling rate of motor units.^116^ In the context of force control, however, the potential positive effects of lifelong physical activity have yet to be investigated. Indeed, our current perception of the relationship between aging and force control is based on studies comparing heterogeneous groups of sedentary to moderately active older adults with young adults.^23^ As such, how much of the age‐related decrement in force control is mediated by an inherent aging process or aging interacting with the deleterious effects of sedentary behavior is unknown. Moreover, the mechanisms underlying the decrement in force control may be more related to inactivity compromised physiology, rather than simply age, or most likely an interaction of the two.^113^

Given the maintenance of other aspects of physiological and muscle function in physically active adults, the lack of research on lifelong physical activity and force control is a pertinent issue. It is, therefore, imperative that future research on aging and force control seeks to establish whether differences in force control exist between lifelong physically active adults and age‐matched sedentary individuals (i.e., the type of population used in studies to date). Such research will, for the first time, elucidate the effects of aging on force control, and the mechanisms underlying the loss‐of‐force control, independently from those of inactivity. Moreover, tracking lifelong physically active older adults over a number of years, alongside age‐matched controls, would be novel and provide insight into the inherent aging process.^117^

A further area of focus for future research is the type of training intervention used in longitudinal studies. Given that muscle strength is not responsible for the age‐associated loss‐of‐force control and that training‐induced improvements in force control can occur independently of increases in strength,^89^ strength training might not be the most appropriate or effective choice of intervention. Accordingly, specific force control training (consisting, for example, of tracking an oscillating target^118^) is an intervention that should be given consideration. Indeed, such training has been demonstrated to be superior to strength training at increasing force steadiness and, importantly, gait variability in stroke survivors.^119^

## 8 PERSPECTIVE

There has, in recent years, been increasing research interest into how and why muscle force control decreases with age. Such research has demonstrated that the age‐associated loss of muscle force control is not only characterized by an increase in the magnitude of force fluctuations but also by a loss of complexity in force fluctuations. Importantly, this loss‐of‐force control is predictive of poorer performance of the fundamental motor skills, that is, balance, locomotion and manual dexterity, inherent to activities of daily living. Recent research has provided the strongest evidence yet for the mechanistic basis of the age‐associated loss‐of‐force control: namely, an increase in common synaptic input to motor neurons across the lifespan. There are, however, still many unanswered questions relating to aging and force control and, as such, there is a tremendous opportunity to perform studies that determine: (1) the source of the age‐related increase in common synaptic input to motor neurons; (2) whether physical activity interventions to reverse age‐associated changes in force control can also influence functional performance; and (3) whether lifelong physical activity has a protective role against the age‐associated loss‐of‐force control.

## FUNDING INFORMATION

The authors received no funding for this work.

## CONFLICT OF INTEREST

The authors report no conflict of interest.

## Data Availability

Data sharing is not applicable to this article as no new data were created or analyzed in this study.
